# Achieving Healthcare Equity: Disparities in Diagnosis, Quality of Care, and Outcomes Among People With Dementia

**DOI:** 10.1093/geroni/igaa057.2303

**Published:** 2020-12-16

**Authors:** Maricruz Rivera-Hernandez, Amit Kumar, Amit Kumar

## Abstract

Alzheimer’s disease and Related Dementia (ADRD) is a significant public health problem and improving the quality and efficiency of care for older adults with ADRD is a national priority. Approximately five million older adults in the United States, including 50% of nursing home residents and 20% of community-dwelling elderly, have ADRD or probable dementia. Although, the number of minorities affected by ADRD growing at an alarming rate, the diagnosis of ADRD and supportive care for this condition are more likely to be delayed among racial/ethnic minority groups. Given the need to ensure equity of care among racial and ethnic groups, there is a pressing need to understand disparities in diagnosis, access and quality of care among racial and ethnic groups with ADRD, specifically using nationally representative data. This symposium will feature four presentations that provide novel insight regarding racial disparities among people with ADRD in the community-, institution-based post-acute, and long-term settings. Individual presentations will describe 1) racial and ethnic differences in risk and protective factors of dementia and cognitive impairment without dementia; 2) racial and ethnic disparities in high-quality home health use among persons with dementia; 3) Within- and between-nursing homes racial and ethnic disparities in resident’s outcomes for people with ADRD; and 4) racial differences in transition to post-acute care and rehab utilization following hip fracture related hospitalization in patients with ADRD. Finally, there will be a discussion regarding policy and clinical implications, as well directions for future research.

